# Mitochondrial links between brain aging and Alzheimer’s disease

**DOI:** 10.1186/s40035-021-00261-2

**Published:** 2021-09-01

**Authors:** Heather M. Wilkins, Russell H. Swerdlow

**Affiliations:** 1 University of Kansas Alzheimer’s Disease Research Center, Kansas City, KS USA; 2 Departments of Neurology, University of Kansas Medical Center, Kansas City, KS USA; 3 Departments of Biochemistry and Molecular Biology, Medical Center, University of Kansas Medical Center, Kansas City, USA; 4 Departments of Molecular and Integrative Physiology, Medical Center, University of Kansas Medical Center, Kansas City, KS USA

**Keywords:** Aging, Alzheimer’s disease, Mitochondria, Mitochondrial DNA

## Abstract

Advancing age is a major risk factor for Alzheimer’s disease (AD). This raises the question of whether AD biology mechanistically diverges from aging biology or alternatively represents exaggerated aging. Correlative and modeling studies can inform this question, but without a firm grasp of what drives aging and AD it is difficult to definitively resolve this quandary. This review speculates over the relevance of a particular hallmark of aging, mitochondrial function, to AD, and further provides background information that is pertinent to and provides perspective on this speculation.

## Background

Higher organisms invariably age. For those moving forward from life’s beginning, aging may manifest as physical growth and the acquisition of new abilities. For organisms approaching the end of life aging more typically manifests as increased frailty, disability, and risk of death. Investigators who study the biology of aging, especially its later life trajectory, identify “hallmark” biological features that associate with aging, and may contribute to the aging process [1].

Aging clearly alters people’s lives independent of disease, although we do not classically view aging-driven morbidity with the same urgency as disease-driven morbidity. In some cases, these morbidities overlap qualitatively and arguably differ mostly on a quantitative level. For example, with advancing age the ability to retain new information declines in humans, but until relatively recently the medical field has assumed that limited cognitive decline in elderly individuals  does not imply disease and referred to this phenomenon as age-associated cognitive decline (AACD) or age-associated memory impairment (AAMI) [2]. Currently, however, medical practitioners recognize that although not all cognitive changes that occur with aging represent AD or will evolve to AD, these are not necessarily benign syndromes [3, 4]. When manifesting in the absence of objective cognitive deficits they are now sometimes preferably characterized as subjective cognitive decline (SCD) [5], and as mild cognitive impairment (MCI) when objective cognitive deficits are present in the absence of frank dementia [6].

The realization that subtle cognitive change often progresses over time to become functionally consequential has helped change the initial perspective. Moreover, the brains of individuals with SCD and MCI frequently contain plaques and tangles, histologic features used to define Alzheimer’s disease (AD). This complicates the question of where to draw the line between AACD, AAMI, SCD, MCI and AD. One way to simplify this decision, as proposed by Jack et al., is to think of it as AD, or at least as an “AD continuum” when plaques are present [7] and to think of it as aging when they are not.

This approach could make sense if amyloid plaques are in fact the proximal cause of AD, and additionally if the biology of amyloidosis and the biology of aging are completely independent. Currently we do not know the answer to either of these points, but there are reasons to suspect neither is the case. In fact, to understand AD what we truly need is a reliable mechanistic definition of AD. Linking aging mechanisms with AD mechanisms can help inform the development of an AD mechanistic definition. To this end, mitochondria, which are implicated in both aging and AD [8], warrant consideration.

### Mitochondria: leading the fight against entropy

Organisms need energy to maintain form and function. Without energy, entropy overwhelms life. The need to acquire energy and consume it is a critical factor that underlies billions of years of evolution. The procurement of energy from the environment, initially from fermentable substrates and then the sun, drove the creation of all life’s building blocks. In some cases, this success in turn altered the environment; the earth’s current oxygen atmosphere is essentially a direct consequence of photosynthesis. A changing environment in turn prompted additional evolution, including the emergence of mitochondria specialized to capture energy stored in covalent bonds by using it to generate high-energy electrons, harvesting that electron energy in the respiratory chain, and delivering the spent electrons to oxygen.

Eukaryotic cells arose partly through evolutionary advances in energy biology that prompted the emergence of multicellularity. Multicellularity itself developed to efficiently provide energy substrates to individual cells, which could henceforth work together to their advantage. The brain itself is perhaps the ultimate culmination of this process.

Circulation systems developed to deliver energy substrates to cells present in emerging tissues and organs, and to remove byproducts of energy metabolism. The circulatory system brings oxygen and carbon fuels to all parts of the body and carries off carbon dioxide and in some cases newly generated carbon molecules. Without the need to support energy acquisition and production, there is perhaps no need for a circulatory system. For many cell types, including neurons, mitochondria are responsible for laundering energy acquired from external sources to forms they can utilize.

Other reviews have detailed the intricacies of mitochondrial function. Briefly, organic molecules including fatty acids, amino acids, carbon-based molecules derived from fat, and carbon-based molecules derived from glucose enter the organelle. Enzymes within the mitochondria break down those molecules and direct the newly generated carbon pieces elsewhere. Energy released during this process drives redox reactions that feature the reduction of the oxidized form of nicotinamide adenine dinucleotide (NAD+) to NADH and flavin adenine dinucleotide (FAD) to FADH2. NADH and FADH2 can donate their thusly acquired, relatively high-energy electrons to a respiratory chain that gradually milks the energy from those electrons to pump protons from one side of a membrane to another. This creates electrochemical and pH gradients across the membrane. Energy released during a physiologic discharge of these gradients is captured in the form of the phosphate bond that defines the conversion of ADP to ATP. The spent electron is ideally disposed of by the enzyme cytochrome oxidase (COX; complex IV), which passes 4 electrons at a time to molecular oxygen (O_2_) and protons to generate water.

Other reviews have detailed different aspects of mitochondrial biology. These include mitochondrial biogenesis, the process through which cells renew their mitochondria or expand the amount of mitochondria they contain [9]; mitophagy, the process through which cells remove mitochondria or parts of mitochondria [10]; mitochondrial fission and fusion, which allows mitochondria to either discard spent parts or to function in a complementary fashion [11]; and mitochondrial movement, which distributes mitochondria to different parts of cells [12].

In addition to a central role in cell energy production, mitochondria are also a critical source for macromolecule synthesis. For example, carbon entering the Krebs cycle as acetyl CoA can exit the mitochondria at the citrate intermediate step, and in the cytosol that citrate is subject to an ATP-dependent lyase reaction that releases an acetyl CoA, which is used to synthesize lipid molecules. Overall, in this case the mitochondria determine the ultimate destination of glucose-derived carbon, which ends up as lipid carbon.

Mitochondrial physiology plays a role in other cell physiologies, including oxidative stress, calcium homeostasis, iron homeostasis, phospholipid synthesis, cell death, protein trafficking, and proteostasis. They alter multiple signaling cascades. Mitochondria communicate to the nucleus through a process called retrograde signaling [13], which provides information on the state of the mitochondria specifically and the state of the cell in general to the nucleus.

The communication between mitochondria and the nucleus is particularly important as part of the mitochondrial proteome is encoded by DNA contained within the mitochondria itself, the mitochondrial DNA (mtDNA), while the rest of the mitochondrial proteome is encoded by nuclear genes that are translated outside of the mitochondria and the newly synthesized proteins are subsequently transported into the mitochondria. Mitochondrial proteomes differ between different cell types [14, 15], consistent with the observation that mitochondria in different cell types may emphasize different cell contributions. For instance, neuron mitochondria lack the enzymes required to perform fatty acid β-oxidation, while astrocyte mitochondria contain those enzymes [16]. This keeps neurons from consuming fatty acids, which is potentially advantageous for maintenance of extensive membrane, while possibly allowing astrocytes to generate ketone bodies that can subsequently transfer to neurons and support neuron oxidative phosphorylation [17]. A similar relationship is apparent when it comes to glucose utilization. Astrocytes can exploit aerobic glycolysis to produce lactate, which can subsequently transfer to neurons where it supports neuron oxidative phosphorylation [18].

### Mitochondrial aging, mitochondria in aging

There is consensus that mitochondria change with advancing age [19]. Studies have demonstrated age-related declines in mitochondrial mass, respiration capacity, and respiration efficiency. Such changes appear to span multiple tissues. Many investigators, but not all, have reported a relative increase in the burden of heteroplasmic mtDNA mutations [20, 21], which manifest as both point mutations and deletions. Surprisingly, the relationship of mtDNA copy number with aging is unclear as the literature reports findings of increased, unchanged, or decreased mtDNA copy number with aging [22]. Although the discrepancy can be partly explained by the use of different tissues for measurement, which has yielded different results, there are indeed inconsistencies among different studies on the mtDNA copy number change in a particular tissue. Model utilization and methodologic factors presumably contribute to this inconsistency [23].

Other biological parameters that are related to mitochondria also change with age. Oxidative stress is a well-described parameter that increases with advancing age [24]. This manifests as increased DNA oxidation, and in general, with advancing age mtDNA oxidation accumulates more rapidly than nuclear DNA (nDNA) oxidation [25].

In many species, especially in humans, some potential confounders should be considered. For example, older individuals are likely more sedentary than younger individuals. This could lead to muscle deconditioning, which may in turn affect muscle mitochondria. Also, many tissues contain more than one type of cell and this could influence the outcomes. Consider the case of blood, which contains red blood cells, white blood cells, and platelets. The mature red blood cells predictably lack nDNA and mtDNA, and platelets contain mtDNA but not nDNA. The white blood cells contain both. Since the mtDNA copy number determination frequently relies on the measured mtDNA-to-nDNA ratio in a sample, the extent of enrichment of the white blood cells relative to the platelets in the assay can have a critical impact. This scenario also applies to the brain. Levels of neuron and astrocyte mtDNA may differ, as may age-related neuron and astrocyte changes. Separating neurons and astrocytes requires a concerted effort, and even when applied, such procedures can produce different degrees of cell-type enrichment.

The nDNA also contains mtDNA pseudogenes, referred to as nuclear-mtDNA (NUMT) sequences, which can influence the mtDNA-to-nDNA ratio determinations. The procedure used to harvest DNA from cells is also consequential, as some approaches may preferentially exclude mtDNA from the assayed sample and thereby skew the true mtDNA-to-nDNA ratio [23].

Whether the age-associated changes to mitochondria are a consequence of aging or drive aging is unclear. Classic mechanistic-oriented aging hypotheses, such as the free radical theory of aging, have been formulated from the perspective that mitochondria drive aging [26, 27]. The free radical hypothesis speculates that free radicals generated by the mitochondrial respiratory chain oxidize cell molecules to the point of perturbing their function over time. Some investigators went a step further to propose a particular molecule that might accumulate longitudinal damage and manifest a progressive functional decline, thereby serving as an aging “clock” [28]. The observation that levels of mtDNA somatic mutation rise with increasing age supports the speculation that mtDNA might constitute that clock [20, 29].

To better assess the cause versus consequence question, investigators turned to or developed animal models. As might be expected, such studies established valuable precedents while raising further questions. One influential model is the mtDNA mutator mouse, which features a proofreading mutation of the mtDNA polymerase gamma that mediates mtDNA replication [30, 31]. These mice acquire mtDNA mutations at an accelerated pace and manifest phenotypes that are consistent with accelerated aging. This would seem to resolve the issue of whether mtDNA-derived changes in mitochondrial function can drive aging, but some cautions should be taken against extrapolating conclusions from the mutator mice to human aging [32–34]. The homozygous mutants, which demonstrate accelerated aging phenotypes, accumulate levels of mutation that far exceed levels found in aging humans. The heterozygous mutants also accumulate higher levels of mtDNA mutation than those found in aging humans, but do not show accelerated aging phenotypes. In addition, neither the homozygous nor the heterozygous mutants show evidence of oxidative stress, which argues against the role of free radicals as a requisite intermediary.

Different models have also generated, at least at a superficial level, conflicting results. While the mtDNA mutator mouse data suggest that free radicals do not impact aging [35, 36], in some cases the increased expression of enzymes that counter mitochondrial oxidative stress prolongs longevity in mice [37]. Further complicating the picture, some models designed to or some interventions intended to enhance mitochondrial function show extended lifespan, while some models designed or interventions intended to interfere or reduce mitochondrial function also paradoxically show extended lifespan [38].

**Fig. 1:**
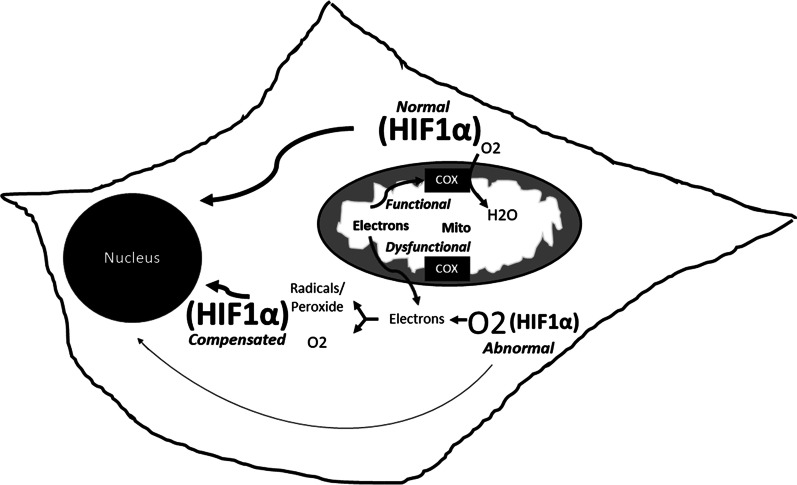
Oxidative stress helps modulate HIF1α levels. In the cell depicted, when the mitochondrial respiratory chain functions appropriately, O_2_ is consumed at COX, which keeps O_2_ from accumulating and creates a HIF1α setpoint. With mitochondrial respiratory chain failure, intracellular hyperoxia can develop, with a consequent reduction in HIF1α. The respiratory chain dysfunction can cause electron egress from the mitochondria and subsequent production of free radicals and hydrogen peroxide, which can boost HIF1α levels by relieving hyperoxia or by accomplishing HIF1α stabilization. The thickness of the line leading from HIF1α to the nucleus is intended to confer greater versus lesser amounts of HIF1α signaling within the cell. Mito, mitochondria

A deeper understanding of the myriad changes that arise in these simplified models, and the reasons underlying these changes, will hopefully eventually resolve at least some of these apparent conflicts. Reactive oxygen species (ROS), for instance, may alternatively harm or protect cells. The classic thinking that ROS serves mostly to damage lipids, DNA, and proteins is being replaced by a more recent appreciation of its physiologic roles, which include cell signaling and the activation of hormetic responses [39]. ROS may also provide cells with a mechanism that allows them to better regulate their oxygen levels. This could prove particularly important in the setting of respiratory chain dysfunction as the respiratory chain is responsible for the majority of cell oxygen consumption. It is worth considering that a primary reduction in mitochondrial respiration could induce a state of intracellular hyperoxia, with protean secondary effects such as a reduction in hypoxia induction factor 1 alpha (HIF1α) signaling. In this case the generation of ROS could conceivably help restore the cell oxygen level to a more physiologic state and consequently normalize HIF1α activity [40]. Figure 1 illustrates this scenario. At least one study performed in *C. elegans* is consistent with this possibility [41]. A comprehensive yet precise understanding of the biological consequences of different mitochondrial manipulations may lead to a better understanding of the mitochondrial-aging nexus.

### Mitochondria in AD

When analyzing groups of mitochondria from persons with AD and from persons who are cognitively normal, on multiple parameters the mitochondria between the two groups do not function equivalently or otherwise appear distinct [42–45]. Some differences may reflect bona fide lesions or pathology, while others likely reflect adaptations or compensations. The altered mitochondria-associated endpoints can be internal or external to the mitochondria themselves. In some instances, differences represent an exaggeration of age-associated changes. In others, the differences are unique.

Of critical importance, mitochondrial alterations are not brain-limited. The differences are also seen in platelets, fibroblasts, and muscle [42, 46–48]. This implies that the alterations are not exclusively a consequence of neurodegeneration, and probably not exclusively caused by the classic AD Aβ-plaque and tau-tangle biomarkers. They are seen in individuals with the MCI syndrome [49], which often manifests as a precursor to AD with dementia.

The age-related reductions in respiratory chain capacity and efficiency are aggravated in AD. The COX activity or related function is lower in AD brain tissues, platelets, and fibroblasts than in age-matched groups [42, 50]. Heteroplasmic mutations that accumulate with advancing age accumulate to a greater degree in AD [25, 51]. The mitochondrial fission–fusion balance shifts towards fission [52, 53], and AD brain and cell models show a relative reduction in mitochondrial movement [54, 55]. Overall, AD brain mitochondria are smaller in size, yet manifest increased numbers of swollen organelles with disrupted cristae [43, 56, 57]. Reductions in peroxisome proliferator-activated receptor gamma coactivator 1 alpha at both protein and mRNA levels suggest reduced mitochondrial biogenesis [58, 59]. Decreased mitophagy has also been reported [60], although one study has noted an increase in autophagosomes containing mitochondrial detritus [61]. Oxidative stress has been consistently reported to increase [62], and it is tempting to link this phenomenon, as well as reduced COX activity, to an apparent reduction in HIF1α signaling [63].

In AD, the HIF1α system seems especially vulnerable to mitochondrial dysfunction. HIF1α is constitutively expressed. However, in the presence of sufficient cell oxygen, a set of prolyl hydroxylase domain-containing enzymes will utilize oxygen, in conjunction with cytosolic α-ketoglutarate and ferrous iron, to hydroxylate HIF1α proline [64]. This proline hydroxylation targets HIF1α for ubiquitination and removal via the proteosome. In this situation, HIF1α is not available to move to the nucleus and cannot drive the expression of its dependent genes. When cell oxygen levels are low, hydroxylation of HIF1α proline cannot occur, and HIF1α is not degraded and thus can promote gene expression.

Through sensing cell oxygen levels and coordinating a response to those levels, HIF1α modulates energy metabolism and its pertinent fluxes. HIF1α signaling promotes glycolysis and restrains mitochondrial respiration [64]. Its action, though, seems designed to help cells respond to environmental oxygen levels. In general, the HIF1α system encourages cells to generate ATP through respiration when oxygen is adequate, and to generate ATP through fermentation when it is not [65]. In the setting of a primary mitochondrial dysfunction, this could present a conundrum. If oxygen levels rise because it is not consumed by the respiratory chain, HIF1α would be down-regulated, and a subsequent reduction in glycolysis could exacerbate bioenergetic stress.

Studies of AD brains have more consistently reported reduced mtDNA than studies of aging brains [66–68], despite a notable increase in autophagosome mtDNA, which contains large amounts of mtDNA deletion [61]. The mtDNA decrease in AD brain may not simply reflect an artifact of reduced neuron number, as a previous study that quantified mtDNA deletions in single neurons reported elevated levels of mtDNA deletions and established that mtDNA in AD neurons is indeed altered [69]. Cerebrospinal fluid (CSF) mtDNA is also lower in AD than in control CSF [70]. One study has reported evidence of a reduction in lymphocyte mtDNA [71].

The anatomically widespread localization of altered mitochondrial function in AD raises the question of its origin. Cytoplasmic hybrid (cybrid) studies argue that factors intrinsic to the mitochondria themselves, especially mtDNA, contribute to this [8]. In these studies, mitochondria from, usually platelets, of AD or age-matched control subjects are transferred to cell lines completely depleted of endogenous mtDNA, or ρ0 cells [72]. The mtDNA contained within the transferred mitochondria repopulates the ρ0 cells and restores the lost mitochondrial functions. The groups of cybrid cell lines generated through the transfer of AD subject platelet mitochondria show persistent differences in mitochondrial function when compared to the cybrid cell lines receiving control subject mitochondria [73]. As these cells have the same nuclear content and are maintained under identical conditions, the difference in mitochondrial function most likely arises from and indicates differences in mtDNA.

Differences between AD and control cybrid lines are subtle but diverse. As is the case in multiple primary tissues, COX activity is lower in the AD subject-generated cybrids [8]. The AD subject-generated cybrids display elevated levels of oxidative stress, a reduced mitochondrial membrane potential, a reduced ability to buffer changes in cytosolic calcium, reduced mitochondrial movement, and a smaller overall size despite an increase in swollen mitochondria with disrupted cristae. Several markers of mitochondrial biogenesis appear lower.

The precise nature of the presumed responsible AD subject mtDNA signature thus far remains unclear. There does not appear to be a single mtDNA variant responsible for these differences. To date, there is no evidence for a heteroplasmic mutation, whether somatically acquired or inherited. Recent studies, however, do reveal that cybrid cell lines containing mtDNA from different haplogroups function differently [74]. This suggests that certain mtDNA variants occurring in combination may play a role. This view has been supported by genetic association studies [75]. The mtDNA control region also demonstrates an impressive degree of diversity between individuals, and some of this control region diversity is haplogroup-specific. Studies of AD brain mtDNA do suggest that some control region variants differ between AD and controls [76]. As the mtDNA control region helps determine mtDNA replication and transcription, it can affect a variety of mitochondrial functions. One cybrid study found a small but consistent decrease in mtDNA copy number in AD subject-generated cybrid lines relative to control subject-generated cybrid lines [77], potentially consistent with the possibility that the mtDNA copy number represents a source of at least some altered mitochondrial functions in AD, and that the lower levels of mtDNA are potentially mediated by inherited combinations of control region variants.

Some aspects of AD mitochondrial function, however, likely arise extrinsic to the mitochondria themselves. Several studies have reported that amyloid precursor protein (APP) and Aβ localize to mitochondria, and Aβ appears to act as a mitochondrial toxin [78–85]. The tau protein also appears to localize to mitochondria and contribute to mitochondrial dysfunction through multiple mechanisms [86–92]. Similarly, a cleavage product from the apolipoprotein E protein displays a mitochondrial localization signal, targets mitochondria, and inhibits COX activity [93, 94]. Due to the differences in protein folding, apolipoprotein E encoded by the APOE4 isoform generates larger amounts of the mitochondrial-targeting cleavage peptide. Recent studies have found that the COX activity in platelet mitochondria of AD subjects is lower in *APOE4* carriers than in *APOE4* non-carriers [95, 96]. Overall, the differences in mitochondrial function may arise at least to some extent from the differences in mtDNA and nuclear genes, a view that can readily explain the systemic AD biochemical and molecular phenotypes. Because mitochondria also influence other biological modules implicated in AD, such as endosomal function, lipid biology, and innate immunity and inflammation [97], there are valid reasons to propose that mitochondria play a primary rather than a secondary role in AD. This idea is captured by the AD mitochondrial cascade hypothesis [50, 98–101].

### Mitochondria: the missing mechanistic link between aging and AD?

If mitochondria represent a common underlying hallmark of both aging and AD, then it is possible that a situation could exist in which during aging, the mitochondrial drivers remain minor enough to permit an adequate functional compensation, whereas in AD adequate compensation is not possible. Under this scenario, “compensated” brain aging can give way to “uncompensated” brain aging. The basis for this is simply the mitochondrial decline progressing past a critical point. A progressive age-related decline in respiratory chain function, for example, could potentially drive such a phenomenon.

In a slightly different but distinct modification of this scenario, a unique mitochondrial change could arise that then triggers the transition from aging to disease. The difference from the scenario described above is that progressive age-related mitochondrial changes come to eventually alter mitochondria in a way not typically observed during compensated aging. By way of example, if it is correct that the mtDNA copy number truly does not fall with advancing age but does during AD, then the point at which mtDNA copy number falls would represent the start of the disease. Ultimately, both these possibilities could play out.

Of course, the question of how the classic AD pathologies relate to any of this is critical to this discussion. Alois Alzheimer described the classic features over a century ago [102, 103], and a series of more recent hypothesis-driven and unbiased “omic” studies have identified subsequent additional ones [104, 105]. Plaques and tangles are seen in older persons independent of clinical changes, and among cognitively intact individuals the likelihood of their presence increases with increasing age [106]. If clinical symptoms define the border between the absence versus presence of AD, then plaques and tangles themselves are age-associated changes and perhaps even driven by aging or whatever it is that drives aging. This would infer that the plaques and tangles, toxic or not, are at least a consequence of an upstream biology that allows for or drives their appearance. Complicating this consideration are recent attempts to redefine AD as simply the presence of plaques and tangles, regardless of clinical status [7], but mechanistic considerations do not inform this convention.

Well-documented links exist between mitochondria and Aβ-related biology. The APP from which Aβ derives contains a mitochondrial targeting sequence [78–80]. APP is seen in mitochondria, where it appears to lodge within the translocase of the outer mitochondrial membrane 40 kD complex. APP has been reported to interfere with COX activity. Aβ itself is found within mitochondria, where it may disrupt the function of several proteins [81, 83, 84]. Perturbing mitochondrial or bioenergetic function, alternatively, alters APP processing, and through this, presumably Aβ generation [107–109]. With respect to understanding the relationships between mitochondria, APP and Aβ, one might first need a better understanding in general of APP’s roles within a cell, and specifically at the mitochondria. It would be helpful to know what factors regulate mitochondrial APP accumulation. The field has already recognized some factors that can link the two biologies to each other. Iron is one example, as mitochondria play a critical role in iron homeostasis, and iron levels also regulate APP expression [110].

Links also exist between mitochondria and tau biology. Tau localization to mitochondria has been reported and it interferes with mitochondrial function through multiple mechanisms [86–92]. Conversely, various mitochondrial toxins increase tau phosphorylation and oligomerization [111–117]. Both chronic and acute mtDNA depletion result in increased cell total tau, increased oligomer tau, and in the case of chronic mtDNA depletion, a relative shift from monomer to oligomer tau [77]. Cybrid cell lines derived from AD subjects on average contain more oligomer tau than cybrid cell lines derived from cognitively normal subjects [77].

**Fig. 2:**
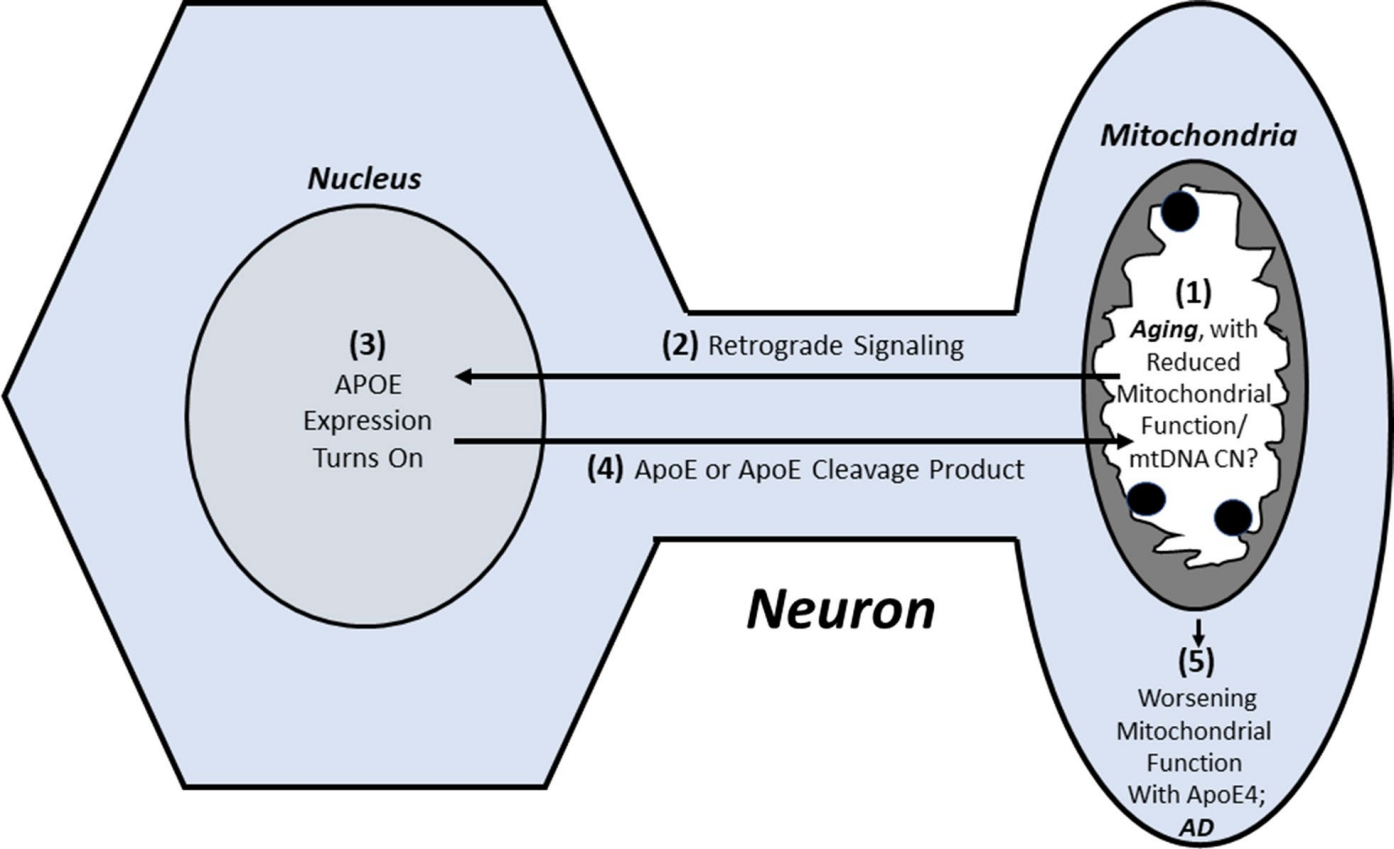
Could age-related changes to mitochondria alter ApoE biology? Neurons do not typically express the *APOE* gene to produce ApoE protein, but may begin to express it in the setting of an age-related decline of mitochondrial function or mtDNA copy number. The ApoE protein that is produced, or a cleavage product of ApoE, may then interact with mitochondria. If the APOE isoform is APOE4, this may confer a toxic effect that further perturbs mitochondrial function and pushes the brain from a state of compensated to uncompensated brain aging, a state equivalent to AD. While this illustration presents a dramatically simplified scenario, it conceptualizes a series of events through which biological changes typically associated with aging could transition into biological changes typically associated with AD

Mitochondria could also potentially provide insight into how apolipoprotein E influences AD risk. Although several hypotheses exist [118], this almost 30-year conundrum remains incompletely resolved. As mentioned in the previous section, one hypothesis is built on the observation that an apolipoprotein E cleavage product, excessively generated from the APOE4 isoform, targets mitochondria and acts as a mitochondrial toxin [93, 94]. As *APOE* gene expression is typically quite limited in neurons and occurs mostly in astrocytes and microglia [119, 120], presumably in this scenario neuronal *APOE* expression must begin to occur. One very recent study reported that this is indeed the case [121]. However, that study fell short of identifying what event might occur in neurons to turn on their APOE production. It would be interesting to test whether altered mitochondrial function can turn on neuron APOE expression. If this in fact happens, one possible mechanism that ties aging to AD could occur as follows: age-related changes in mitochondrial function occur, these changes reach a point that they turn on neuron *APOE* expression, the apolipoprotein E or a cleavage product localizes to the mitochondria to further interfere with mitochondrial function, and the degree of mitochondrial compromise within the cell worsens to surpass the ability of the cell to compensate (Fig. 2). Because APOE4 generates higher levels of the mitochondrial toxin, under this scenario APOE4 would prove relatively detrimental when compared to the other APOE isoforms.

This of course has treatment implications. *APOE* genetics arguably offer therapeutic clues, but how to best leverage apolipoprotein E biology for treatment purposes is a complex issue. On a general level, a loss of function could justify increasing the levels, while a toxic gain of function could justify decreasing the levels. On a more isoform-specific level, some might argue for increasing the levels of the “protective” APOE2 isoform, while others might argue that the best approach is to decrease the levels of the “toxic” APOE4 isoform. Under the scenario described above, a logically directed approach would be to prevent the expression of APOE, regardless of the isoform but especially in the presence of APOE4, within neurons.

### Additional considerations

This review narrowly focuses on the relationships between mitochondria and aging, and between mitochondria and AD, while trying to make a case for how mitochondria could link aging to AD. If this perspective is correct, it may be worth considering whether mitochondria in fact initiate AD [50, 98–100]. This would of course conflict with the current popular hypothesis, the amyloid cascade hypothesis [122], that has strongly influenced AD research for three decades despite robust dissent. Criticisms are leveled from multiple angles, such as the repeated observation that plaque burden sub-optimally correlates with cognitive deficits [123–125], and the more recent appreciation that reducing Aβ, by preventing production or promoting removal, will at best minimally benefit patients [126]. Partly for these reasons, interest in other etiologic hypotheses has emerged. This includes an emerging interest in tau [127], whose fibrillar presence in the form of tangles quantitatively correlates well with cognitive decline [128].

It is also important to recognize that AD is divided into subtypes whose etiologies may or may not overlap. A prominent distinction exists between late-onset AD (LOAD), which does not show a classic Mendelian inheritance pattern, and early-onset familial AD, which does [106]. Advancing age would most obviously prove pertinent to LOAD. However, if the role of deterministic mutations is to reduce the amount of age-related mitochondrial decline needed to initiate clinical disease, for example by retarding mitochondrial resilience or the brain’s ability to tolerate age-related mitochondrial dysfunction, it is conceivable that age-related mitochondrial changes could contribute to early-onset familial AD.

## Conclusions

To advance our understanding of the aging–AD nexus, we must first overcome several challenges. On the one hand, aging is a complex process that implicates multiple contributing factors, any of which alone or in combination could prove pertinent to AD. On the other hand, despite remarkable advances in our ability to detect AD pathologies in living individuals, our understanding of what drives these pathologies remains incomplete.

The new biomarker-based “biological” AD definition [7], to some extent, also presents a challenge. Intentionally or not, it creates an arbitrary divide that conceptually separates aging from AD as aging with plaques and tangles is simply designated AD. While this approach may facilitate the standardization of participant groups entering clinical trials, equating biomarkers with disease does not necessarily explain why those biomarkers arose or how they got there. Arguably what the field really needs to develop is a “mechanistic” definition of AD. To this point, the remarkable positive correlation of advancing age with AD incidence and prevalence can provide clues on where to look [106]. Both aging and AD feature commonalities in mitochondrial biology, and mitochondrial biology interacts with most of the accepted AD pathologies and pathways [8, 98]. Pursuing mitochondrial links between aging and AD may, therefore, lead to a better understanding of AD, which could lead to better interventions.

## Data Availability

Not applicable.

## Abbreviations

Aβ: Beta amyloid
AACD: Age-associated cognitive decline
AAMI: Age-associated memory impairment
AD: Alzheimer’s disease
APP: Amyloid precursor protein
COX: Cytochrome oxidase
CSF: Cerebrospinal fluid
Cybrid: Cytoplasmic hybrid
FAD: Flavin adenine dinucleotide
HIF1α: Hypoxia induction factor 1 alpha
LOAD: Late-onset Alzheimer’s disease
MCI: Mild cognitive impairment
mtDNA: Mitochondrial DNA
nDNA: Nuclear DNA
NAD: Nicotinamide adenine dinucleotide
ROS: Reactive oxygen species
SCD: Subjective cognitive decline
